# Murine Typhus in Austin, Texas, USA, 2008

**DOI:** 10.3201/eid1603.091028

**Published:** 2010-03

**Authors:** Jennifer Adjemian, Sharyn Parks, Kristina McElroy, Jill Campbell, Marina E. Eremeeva, William L. Nicholson, Jennifer McQuiston, Jeffery Taylor

**Affiliations:** Centers for Disease Control and Prevention, Atlanta, Georgia, USA (J. Adjemian, S. Parks, K. McElroy, M.E. Eremeeva, W.L. Nicholson, J. McQuiston); Texas Department of State Health Services, Austin, Texas, USA (S. Parks, J. Taylor); Austin/Travis County Health Department, Austin (J. Campbell); 1 These authors contributed equally to this article.; 2 Current affiliation: Federal Bureau of Prisons, Washington, DC, USA.

**Keywords:** Murine typhus, Rickettsia typhi, emergence, opossums, fleas, zoonoses, bacteria, Texas, research

## Abstract

In August 2008, Texas authorities and the Centers for Disease Control and Prevention investigated reports of increased numbers of febrile rash illnesses in Austin to confirm the causative agent as *Rickettsia typhi*, to assess the outbreak magnitude and illness severity, and to identify potential animal reservoirs and peridomestic factors that may have contributed to disease emergence. Thirty-three human cases of confirmed murine typhus were identified. Illness onset was reported from March to October. No patients died, but 23 (70%) were hospitalized. The case-patients clustered geographically in central Austin; 12 (36%) resided in a single ZIP code area. Specimens from wildlife and domestic animals near case-patient homes were assessed; 18% of cats, 44% of dogs, and 71% of opossums had antibodies reactive to *R. typhi*. No evidence of *R. typhi* was detected in the whole blood, tissue, or arthropod specimens tested. These findings suggest that an *R. typhi* cycle involving opossums and domestic animals may be present in Austin.

## Abstract 2

Physicians should be alert for possible cases in this area.

Murine typhus, also known as endemic or flea-borne typhus, is caused by *Rickettsia typhi*, a gram-negative, obligate intracellular bacillus. This zoonotic disease is primarily maintained in rodent–flea cycles and is transmitted to humans when infected flea feces contaminate the flea feeding site or other skin abrasions (*1*). After an incubation period of 6–14 days, a nonspecific febrile illness may develop with symptoms of headache, arthralgia, abdominal pain, and confusion. Approximately 50% of patients also report the development of a diffuse macular or maculopapular rash, which starts on the trunk and spreads peripherally (sparing the palms and soles) nearly 1 week after the initial onset of fever and can last from 1 to 4 days. Although the disease is easily treated with doxycycline, it can be severe or even fatal if not diagnosed and treated properly (*2**,**3*).

Throughout its global distribution, *R. typhi* has been primarily concentrated in coastal urban areas where it is maintained among rats (*Rattus* spp.) and oriental rat fleas (*Xenopsylla cheopis*) (*3*). Within the United States, murine typhus is endemic in parts of California, Hawaii, and Texas, where <100 cases are reported annually (*4**–**7*) with a 1%–4% fatality rate when left untreated (*3**,**4*). Recent studies in southern Texas and California indicate that the classic rodent-flea cycle of *R. typhi* has been augmented in these suburban areas by a peridomestic cycle involving free-ranging cats, dogs, opossums, and their fleas (*1**,**6**,**7*). In addition, *R. felis*, which may produce a febrile illness in humans (*8*), may also circulate within these same zoonotic cycles (*7**,**9*). Although both agents have been documented in opossum-flea cycles in parts of southern Texas (*7**,**9*), these diseases are rare in the Austin/Travis County area. Though Austin is only 140 km from the Texas coast, where murine typhus is endemic, only 4 cases have been reported there in the past 25 years; 2 of those 4 cases were reported in September 2007 (Texas Department of State Health Services [TDSHS], unpub. data).

From March through July 2008, the Austin/Travis County Department of Health and Human Services (ATCDHHS) identified 13 cases of febrile illness, half of which had a rash or a severe headache, or both. Laboratory tests conducted at the TDSHS and the Centers for Disease Control and Prevention (CDC) indicated that these patients all had antibodies reactive to *R. typhi*. Active infection with *R. typhi* was also identified in 1 patient by PCR. In August 2008, TDSHS, CDC, and ATCDHHS initiated a detailed epidemiologic investigation to confirm the causative agent as *R. typhi*, to assess the outbreak magnitude and illness severity, and to identify potential animal reservoirs and peridomestic factors that may have contributed to disease emergence.

## Methods

In August 2008, TDSHS, CDC, and ATCDHHS initiated an epidemiologic investigation into the emergence of murine typhus in Austin. A clinical investigation was conducted to assess the magnitude and severity of the outbreak. An environmental investigation was conducted to assess the environment and peridomestic factors and domestic animals around case-patient home sites to identify possible means of transmission and risk factors for disease.

### Clinical Investigation

Healthcare providers in Austin were asked to report any suspected cases to the health department. Suspected cases were reported to ATCDHHS by the National Electronic Disease Surveillance System. Criteria for suspected cases were high fever (>38°C), with at least 1 of the following: headache, rash, or myalgia. Confirmed cases were defined as meeting the suspected case criteria and having laboratory confirmation for *R. typhi* infection. The criteria for laboratory confirmation included at least a 4-fold rise in antibody titer to *R. typhi* antigen between paired serum specimens obtained >3 weeks apart or the detection of *R. typhi* DNA in a clinical specimen by PCR.

All suspected and confirmed case-patients identified from March through November 2008 were interviewed in-person or by telephone, medical chart reviews were conducted, and serum specimens were collected for laboratory testing. Where the patient was <18 years old, the parents were interviewed. All patients or their proxies were interviewed by using a standard questionnaire. Information collected included demographics, laboratory test results, and clinical symptoms. Medical records of all patients were reviewed. Abstracted data included results of radiographs, urinalyses, blood counts, serologic analysis, and liver enzyme analyses.

### Environmental Investigation

Environmental assessments were conducted at the households of 21 case-patients who had been identified from March through July 2008. An external site assessment of the physical property was conducted, including evaluations of environmental factors such as housing structure, vegetation, water features, food sources, and evidence of animals present. When possible, household owners were queried on the internal and external use of pesticides, ownership of domestic animals, use of flea- and tick-control products, history of flea infestations, and reported past evidence of rodents or other types of wildlife in or around the property.

Serum and whole blood specimens were collected from cats and dogs from consenting case-patient households, as well as from feral cats submitted by humane organizations working in the area. A total of 791 trap nights using a combination of live traps (H.B. Sherman Traps, Tallahassee, FL, USA, and Tomahawk Live Trap Co., Tomahawk, WI, USA) were also conducted around 10 case-patient households, targeting capture of peridomestic small wild mammals. In addition, wildlife was accepted from organizations that trapped so-called nuisance species within the outbreak area. Wildlife species were released after specimen collection, except for rats, which were humanely euthanized. Serum and whole blood, as well as ectoparasites, were collected from all animals. Tissue specimens (heart, lung, kidney, spleen and liver) were collected from animals that were euthanized. The address of residence or location was recorded for each animal assessed.

### Laboratory Analyses

Confirmatory tests for suspected human cases were performed at a variety of private commercial laboratories; results were then verified by subsequent testing at the TDSHS Laboratory, Austin, Texas, USA, the Rickettsial Zoonoses Branch Diagnostic Laboratory at CDC, Atlanta, Georgia, USA, or both. All animal and arthropod samples were tested at CDC.

#### Serologic Analysis

Serologic analysis was conducted by using indirect immunofluorecent antibody (IFA) assays for *R. typhi* grown in embryonated chicken yolk sacs, air-dried, and acetone-fixed onto template slide wells. In each assay, antibodies bound to the antigens are detected by using species specific fluorescein isothiocyanate (FITC)–labeled conjugates. We used FITC conjugates (Kirkegaard & Perry Laboratories, Gaithersburg, MD, USA) produced in goats against human immunoglobulin (Ig) G (γ-chain–specific at a final dilution of 1:150), human IgM (μ-chain–specific at a final dilution of 1:100), rat IgG (heavy plus light [H + L] chain) (diluted at 1:100), mouse IgG (H + L chain) (1:100), cat (H + L chain) IgG (1:100), and a monovalent conjugate against dog IgG (γ-chain–specific) (1:150). FITC-labeled conjugate against opossum IgG (H + L chain) (Bethyl Laboratories, Montgomery, TX, USA) was used at a final dilution of 1:100. The assay format, buffers, and other reagents were used according to the method described by Nicholson et al. (*10*). Samples were serially (*2*) diluted and the last well demonstrating specific fluorescence of the *R. typhi* organisms was recorded as the endpoint titer (expressed as a reciprocal of the dilution).

#### Amplification by PCR and Sequencing

Fleas were identified to species, and DNA was isolated from each specimen by using the Biomek 2000 Laboratory Automation workstation (Beckman, Fullerton, CA, USA) and reagents from the Wizard Prep kit (Promega, Madison, WI, USA) (*11*). Detection of *R*. *felis* and *R*. *typhi* DNA was conducted by using a TaqMan assay for the citrate synthase (*glt*A) gene of *Rickettsia* spp. as described elsewhere (*11**,**12*). The reactions were conducted by using the Brilliant Q PCR core reagent kit (Stratagene, La Jolla, CA, USA) and run on an *i*Cycler (Bio-Rad, Hercules, CA, USA). Primers and probes were produced by the CDC Core Facility (Atlanta, GA, USA). For animal and human specimens, DNA was extracted from 200 μL of EDTA-blood and 25–50 mg tissue by using the QIAamp DNA Mini Kit (QIAGEN, Valencia, CA, USA). Animal specimens were tested by *glt*A TaqMan. PCR assays for the rickettsial 17-kDa antigen gene were used for detection of spotted fever and typhus group rickettsiae DNA in clinical specimens with Ready-to-Go-Beads (Amersham Biosciences UK Ltd., Little Chalfont, UK) as described elsewhere (*13**,**14*). Amplicons were purified using Wizard SV Gel and PCR Clean-Up System according to the manufacturer’s instructions (Promega). The purified product was sequenced with the ABI PRISM BigDye Terminator Cycle 3.1 Sequencing kit (Applied Biosystems, Foster City, CA, USA). The sequenced product was then purified with a QIAGEN DyeEx 2.0 kit (QIAGEN) and run on an Applied Biosystems 3100x Sequencer (Applied Biosystems).

## Results

### Clinical Investigation

**Figure 1:**
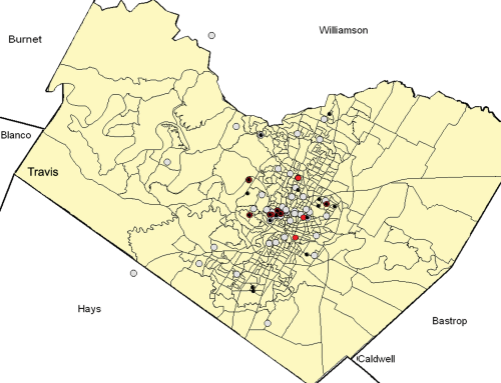
Month of illness onset for laboratory-confirmed murine typhus cases (n = 33) reported in Austin/Travis County, Texas, USA, 2008.

Thirty-three of 53 patients with suspected cases were confirmed to have murine typhus. All 33 were laboratory confirmed by IFA assay; 1 case was serologically confirmed by PCR, and the sequenced product was positive for *R. typhi* DNA. Illness onset among the patients ranged from March through October 2008, with 70% occurring during May–August (Figure 1). Patients with confirmed cases had an average age of 39 years (range 7–64 years, 15% <18 years); most were male (56%) and white (97%). Although no deaths were attributed to murine typhus among this cohort of case-patients, 23 (70%) were hospitalized (mean 7 days; range 3–14 days), and 9 (27%) were admitted to intensive care units (mean 5 days, range 1–10) with complications, including pneumonia, coagulopathy, and renal failure. Seventeen (51%) patients received antimicrobial drugs, 13 (76%) of them doxycycline. The mean time from symptom onset to antimicrobial drug treatment was 8.3 days (median 8 days, range 1–19 days). No significant differences were detected in rates of hospitalization (p = 0.78) and complications (p = 0.84) between those patients who did and those who did not receive doxycycline.

The median high temperature reported among confirmed case-patients was 40°C (range 39°C–41°C). The most commonly reported symptoms included malaise (76%), headache (73%), chills (61%), and myalgia (61%). Loss of appetite (58%), nausea (52%), rash (46%), vomiting (42%), and diarrhea (36%) were also reported by many case-patients. Less than one third of case-patients reported photophobia (30%), arthralgia (33%), stiff neck (24%), backache (21%), abdominal pain (21%), coughing (18%), jaundice (18%), lymphadenopathy (15%), conjunctivitis (12%), and confusion (12%). Serologic results showed that impaired liver function was common in patients (70%), and some had impaired kidney function (21%).

**Figure 2:**
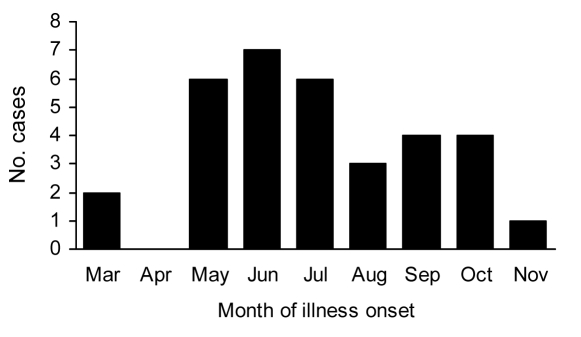
Distribution of confirmed murine typhus case-patients and animals by serologic status for antibodies to *Rickettsia typhi* in Travis County, Texas, USA, 2008. Red circles, confirmed case-patients; black circles, sereopostive animals; gray circles, seronegative animals.

The 33 confirmed case-patients clustered geographically in central Austin (Figure 2). Twelve (36%) resided in 1 ZIP code area in a suburban-residential area (Table 1). Most other patients were from adjacent and nearby central and east central Austin areas. One case-patient resided in northern Travis County but worked in central Austin.

**Table 1:** Distribution by ZIP code of confirmed human murine typhus case-patients and animals and ectoporasites that were tested for *Rickettsia* *typhi* by IFA assay and/or PCR, Austin/Travis County, Texas, USA, August 2008*

ZIP code	No. human case-patients, n = 33	Opossum (12/17)†	Raccoon (0/9)†	Rat (0/4)†	Cat (3/17)†	Dog (4/9)†	Flea (0/139)†
78702	1	1/1	–	–	–	–	0/14
78703	12	6/7	0/5	0/4	0/2	0/3	0/58
78704	1	–	–	–	–	–	–
78705	5	–	–	–	–	–	–
78722	2	–	–	–	3/4	0/1	0/6
78723	1	0/2	–	–	0/3	1/2	0/15
78727	1	–	–	–	–	–	–
78728	1	–	–	–	0/2	–	0/5
78731	1	–	–	–	–	–	–
78741	0	–	0/1	–	–	–	0/2
78745	0	–	0/1	–	–	–	0/4
78746	2	5/7	0/2	–	0/2	2/2	0/31
78747	1	–	–	–	–	–	–
78748	0	–	–	–	0/1	–	0/2
78751	2	–	–	–	–	–	–
78757	3	–	–	–	0/1	1/1	0/2
78759	0	–	–	–	0/2	–	–

*IFA, immunofluorescent antibody. †Values are no. positive/no. tested except as indicated.

### Environmental Investigation

Twenty-six (79%) of the 33 confirmed case-patients owned a dog or cat. Of those, 14 (42.4%) reported regularly administering flea/tick preventatives to their pets. However, only 2 patients (5.4%) noted flea bites or exposure in the 2-week period before illness onset. Recent exposure to opossums was reported by 11 (29.7%) of the patients; >20% had been recently exposed to rats, 19% to raccoons, and 5% to mice through both direct and indirect contact.

External site assessments were performed at 20 home sites (representing 21 case-patients). Of the home sites evaluated, 9 (45%) had pet food outside; 9 (45%) had a garden or compost heap; 12 (60%) had outdoor piles of firewood or harborage; 12 (60%) had apparent evidence of rodents (through direct observations or the presence of feces, nests, or burrows); 17 (85%) had outdoor water sources; and 17 had unsecured garbage outside.

A total of 56 animals (including 17 cats, 9 dogs, 17 opossums, 9 raccoons, and 4 rats) (Table 2) and 139 arthropods were obtained; all but 1 opossum was evaluated (Table 3). Overall, 19 (33.9%) of all animals tested were seropositive. This sample included 3 (17.6%) cats, 4 (44.4%) dogs, and 12 (70.6%) oppossums. None of the samples obtained from raccoons or rats were seropositive. Seropositive animals came from 5 ZIP code areas, and 68% of all seropositive animals came from 2 ZIP codes areas where 35% of the human cases were reported (Figure 2; Table 1). All 3 seropositive cats came from 1 capture site, whereas the 4 seropositive dogs were owned by 3 case-patients (2 dogs by a single patient) from 3 ZIP code areas. Seropositive opossums were from 8 capture sites in 3 ZIP code areas. Of the arthropods evaluated, 83.5% were identified as *Ctenocephalides felis*, the cat flea (Table 3). No evidence of either *R. typhi* or *R. felis* DNA was detected in any of the whole blood, tissue, or arthropod specimens tested.

**Table 2:** Frequency and distribution of animals seropositive for *Rickettsia typhi*, Austin/Travis County, August, 2008*

Animal	No. animals tested	No. (%) animals seropositive	IFA assay titer, no. animals								
			<32	32	64	128	256	512	1,024	2,048	>4,096
Cat	17	3 (17.7)	14	0	0	0	0	1	0	0	2
Dog	9	4 (44.4)	5	0	0	0	0	1	2	1	0

Opossum	17	12 (70.6)	5	4	3	1	3	1	0	0	0
Raccoon	9	0	9	0	0	0	0	0	0	0	0
Rat	4	0	4	0	0	0	0	0	0	0	0
Total	56	19 (33.9)	38	4	3	1	3	3	2	1	2

*IFA, immunofluorescent antibody. Seropositivity indicated by titer >32.

**Table 3:** Summary of fleas collected from animals in Austin/Travis County, Texas, USA, August 2008

Animal	No. animals with fleas/total no. animals	Flea species collected	Total no. fleas collected	Frequency of flea species by host animal, %	Infestation index
Cat	12/17	*Ctenocephalides felis*	23	70.6 (n = 12)	1.35
Dog	1/9	*Ctenocephalides canis*	1	11.1 (n = 1)	0.11
Opossum	18/18	*C. felis*	84	100.0 (n = 18)	4.67
		*Pulex irritans*	14	22.2 (n = 4)	0.78
Raccoon	9/9	*C. felis*	8	44.4 (n = 4)	0.89
		*Echidnophaga gallinacean*	1	11.1 (n = 1)	0.11
		*P. irritans*	5	33.3 (n = 3)	0.56
		*Xenopsylla cheopis*	2	22.2 (n = 2)	0.22
Rat	1/4	*C. felis*	1	25.0 (n = 1)	0.25
Total	41/56	–	139	73.2 (n = 44)	–

## Discussion

Murine typhus is a common zoonotic disease in endemic foci of southern Texas, where a mean of 48 cases were reported annually from 1990 through 2006 (*15*). However, before this investigation, murine typhus was not believed to occur commonly in the Austin/Travis County area, and only 2 cases were identified before 2007. This investigation identified 33 patients with laboratory-confirmed cases, nearly 70% of whom were hospitalized from March through November 2008. In addition, 2 murine typhus cases reported in Austin in September 2007 likely represent some of the first cases associated with this emergent focus. These findings represents the first large-scale outbreak reported in Austin/Travis County since eradication efforts were coordinated in this part of Texas in the 1940s (TDSHS, unpub. data).

The clinical features and age distribution of case-patients reported here are similar to those found in case-patients reported in other murine typhus studies (*4*,*16*). Although 70% of the case-patients identified during this outbreak were hospitalized, this percentage is slightly less than what was observed by Taylor et al. (*16*) during a study of 200 cases in Texas from 1980 through 1984, in which 85% of patients were hospitalized and 1% died. Though no deaths were reported during this 2008 outbreak, nearly one third of all patients were admitted to the intensive care unit with complications (including pneumonia, coagulopathy, and renal failure) that demonstrated the severity of illness.

Delaying treatment for murine typhus increases the duration of symptoms and risk for complications (*4**,**17*). Treatment should always be initiated on the basis of clinical and epidemiologic considerations alone without waiting for a laboratory confirmation of the diagnosis. In this outbreak, 48% of patients did not receive treatment with doxycycline, the drug of choice for treatment for rickettsial diseases. The lack of doxycycline administration and the reported lag time of 1 week to nearly 3 weeks between symptom onset and antimicrobial drug treatment experienced by most patients may have been associated with a delay in recognizing that the cases were murine typhus, because of the perception that the disease was not present in Austin. Despite this finding, the difference in hospitalization and complication rates did not appear to be significant between patients with and without proper antimicrobial drug treatment. However, the small sample size may have precluded a robust comparison of these data.

Strong serologic evidence of exposure to rickettsiae was detected among opossum and domestic animal populations in Austin/Travis County. More than one third of all animals tested were seropositive with *R. typhi* antigen. Of particular interest, >70% of opossums tested were seropositive with *R. typhi* antigen. Further studies are needed to determine the specific role that opossums play in the ecology of murine typhus in the Austin area. Exposure to other rickettsiae in the spotted fever group also cannot be excluded, particularly for *R. felis*, which is very common in cat fleas obtained from opossums (*7**,**12*). The serologic findings observed here are similar to what has been observed in studies of disease-endemic regions in southern Texas and California, USA, where opossums are hosts for fleas containing *R. typhi* and *R. felis* (*6**,**7**,**9**,**18*). In Los Angeles, California, and Corpus Christi, Texas, 42% and 25% of opossums were found to be seropositive for *R. typhi*, respectively, although seropositive rats were rarely or never detected (*7**,**9*). These studies have resulted in a reevaluation of the classic urban cycle of murine typhus in suburban disease-endemic areas in the continental United States, where opossums, domestic cats, and cat fleas—and not rodents and their fleas—are considered to be a primary source of infection (*2*).

Although none of the rats in this study were seropositive for *R. typhi*, the small sample size tested (n = 4) limits our ability to draw conclusions regarding the contribution of rats and their arthropods to the dynamics of murine typhus in this area. Additionally, presumptions regarding contributions of various animal species are limited because only serologic findings were positive; active infection with either *R. typhi* or *R. felis* was not detected in any of the samples tested. While none of the fleas were positive for either *R. typhi* or *R. felis* DNA, this result is not entirely unexpected considering the infrequency with which positive fleas were detected in similar studies. For instance, Boostrom et al. (*7*) identified only 3 *R. typhi* and 11 *R. felis* positive fleas out of a sample of 529 from highly endemic parts of southern Texas. Still, *R. feli*s may be circulating within this area because both pathogens appear to be maintained in complex ecologic cycles (*2**,**7*). More specific studies targeting larger numbers of statistically representative domestic animals and wildlife are needed to better discern complicated human-animal-disease dynamics.

Murine typhus may now be established in the Austin/Travis County area and should be considered an ongoing public health threat. Although, the idea that persons have been infected with *R. felis* (which has been previously found to infect a patient in Texas) cannot be totally excluded (*8*). Continued public health education efforts are needed in the Austin/Travis County area regarding the emergence of flea-borne rickettsiosis and the likely risk factors for infection, with an emphasis on avoiding contact with wild animals and controlling fleas on pets and around the home with approved products. Physicians in the area should maintain an increased vigilance in detecting and diagnosing suspected murine typhus cases as well as other rickettsioses, because timely treatment with the appropriate antimicrobial drug therapy is critical for limiting severe outcomes.
